# Health Research and Millennium Development Goals: Identifying the Gap From Public Health Perspective

**DOI:** 10.5539/gjhs.v8n5p1

**Published:** 2015-08-20

**Authors:** Mona I. El Lawindi, Yasmine S. Galal, Walaa A. Khairy

**Affiliations:** 1 Public Health and Community Medicine Department, Faculty of Medicine, Cairo University, Egypt

**Keywords:** millennium development goals, public health department, research plans-trends

## Abstract

Assessing the research output within the universities could provide an effective means for tracking the Millennium Development Goals (MDGs) progress. This analytical database study was designed to assess the trend of research theses conducted by the Public Health Department (PHD), Faculty of Medicine, Cairo University during the period 1990 to 2014 as related to the: MDGS, Faculty and department research priority plans and to identify the discrepancies between researchers’ priorities versus national and international research priorities. A manual search of the theses was done at the Postgraduate Library using a specially designed checklist to chart adherence of each thesis to: MDGs, Faculty and department research plans (RPs). The theses’ profile showed that the highest research output was for addressing the MDGS followed by the PHD and Faculty RPs. Compliance to MDGs 5 and 6 was obvious, whereas; MDGs 2, 3, and 7 were not represented at all after year 2000. No significant difference was found between PH theses addressing the Faculty RPs and those which were not before and after 2010. A significantly lower percent of PH theses was fulfilling the PHD research priorities compared to those which were not after 2010. This study showed a definite decline in research output tackling the MDGS and PHD research priorities, with a non-significant increase in the production of theses addressing the Faculty RPs. The present study is a practical model for policy makers within the universities to develop and implement a reliable monitoring and evaluation system for assessment of research output.

## 1. Introduction

The MDGs are eight international goals with twenty one targets, with a series of measurable health and economic indicators for each target (United Nations, 2000a). They have been formally originated from the United Nations (UN) Millennium Declaration, following the Millennium Summit of UN in 2000 (United Nations, 2000b). All the UN member states at that time and at least 23 international organizations were committed to achieve the MDGs by 2015 (Lomazzi, Laaser, Theisling, Tapia, & Borisch, 2014). However, some countries achieved many goals, while others were not on track to realize any (United Nations, 2014).

The overall goals of the MDGs were intended to improve an individual’s human capabilities, living standards, and the means to a productive life through emphasizing development in 3 main areas: human capital, infrastructure and human rights (social, economic and political) (Millennium Development Goals, 2015). There have been several consultations on the assessment of progress of the MDGs by a number of organizations, some of which have been officially led by governments, while others were driven by non-governmental organizations (NGOs) and private foundations (WHO, 2012, 2013; PROCOSI, 2013). Even if it is obvious that the health sector played a crucial role in the achievement of the MDGs (The World We Want, 2013); only a few MDGs reports have considered the role of PH (Devex and United Nations Foundation, 2015). It is worth mentioning that the national and international research priorities have a mutual effect on the overall expected output and progress of research within countries. PH research output in the form of theses is one of the important research activities at the academic level, however, they are mostly not formally published or indexed in the web-based scientific literature; accordingly classified as a type of grey literature that is difficult to locate and acquire (LaPelle, Luckmann, Simpson, & Martin, 2006; Osayande & Ukpebor, 2012). Despite that grey literature has its academic benefits because it contains data and statistics that could help assessing the MDGs progress (Fatokun & Amusa, 2014); little attention has been paid to its impact especially the submitted theses for different degrees in Universities (Osayande & Ukpebor, 2012). In this context, it’s worth mentioning that PH research is one of the driving forces for certain MDGs achievements as it aims to produce new knowledge to protect and promote people health and guide policy decisions (Lomazzi et al., 2014). Therefore, evaluating PH research output including the submitted theses within the universities could provide an effective means for tracking the MDGs progress.

The Faculty of Medicine of Cairo University is one of the largest medical schools in Africa, Middle East and the Arab region (Faculty of Medicine, Cairo University, 2011). Therefore, producing a high quality translational research is a priority aiming to disseminate the principles of good and safe practice and consequently improving the health status of the population. One of the objectives of the strategic plan for the Faculty through the period from 2011 till 2014 was enhancing multi-disciplinary research at the national and international levels. According to this plan, the research priority areas to be addressed in community based research were the epidemiology of hepatitis, cancer, hypertension, diabetes, obesity, heart diseases and parasitic infestations (Faculty of Medicine, Cairo University, 2011). The PHD within Cairo University have multiple research activities aiming at knowledge generation to help policy makers in developing strategies and policies to promote health and prevent and control health problems in Egypt. Reproductive health (RH) besides prevention and control of three targeted Egyptian health problems namely renal, hepatic and cancer diseases were the research priority areas set as a target for the PHD research output from 2011 till 2014.

Thus, tracing the progress of PH research regarding the academic research theses in relation to the MDGs is of primary importance for obtaining a complete and more objective and comprehensive overview of the MDGs achievements and failures (McCarthy, Harvey, Conceicao, Torre, & Gulis, 2009). To the researchers’ knowledge, there is no data reporting the progress of PH research theses at the Faculty of Medicine, Cairo University, and their degree of compliance with the MDGs. Therefore, this study was conducted to assess the trend of research theses conducted by the PHD, Cairo University during the period 1990 to 2014 as related to the: MDGs, Faculty, and department RPs and to identify the discrepancies between researchers’ priorities versus national and international research priorities.

## 2. Methods

### 2.1 Study Design and Study Period

This is a routine analytical database study targeting all the approved and registered theses in the PHD. The theses registered centrally in the Post graduate Library of the Faculty of Medicine, Cairo University during the period 1990-2014 for both Master and Medical Doctoral (MD) degrees at the department were targeted. Three time periods were assigned for the study according to two major cut-off points namely at launching the MDGs in 2000 and that of the Faculty and department RPs in 2010. The study was a trial to identify the impact of national and international research priorities on the academic researchers’ priorities. The current study was initiated at the 5^th^ of January 2015 and lasted for 6-months to retrieve all available theses since 1990.

### 2.2 Search Strategy

Despite the presence of several web-based databases indexing the scientific theses from the Egyptian universities, we were not able to electronically access our targeted registered theses in the PHD during the period 1990-2014 through the web-based provided sites during our research in year 2015. Therefore, a manual search of the theses registered in the Postgraduate Library of Faculty of Medicine, Cairo University was started on the 5^th^ of January, 2015. The Postgraduate Library is the final station of any thesis approved by the University as a part of fulfillment of Master or MD degree by the researcher; so it is considered as the most authorized, accurate and trusted source of research at the university level. The main search string was any thesis submitted for the PHD, Cairo University during the period 1990-2014. No restrictions were applied on the study site, setting, design or even the nationality of the researcher.

### 2.3 Theses Retrieval and Indexing

All the output of the search process was retrieved manually by the help of the librarian. For the theses conducted in the period 1990 till 2002; retrieval was from the hard copies available in the library, while for those conducted from 2003 till 2014; it was from the soft copies. The theses were coded and indexed according to the following items:

- Type of the thesis: Master or MD
- Year of thesis approval
- Title of the thesis
- Name and affiliation of the researcher

### 2.4 Data Extraction

After revision of the MDGs and the Faculty of Medicine, Cairo University and PHD RPs to be clearly defined for sake of standardization, a checklist was created to chart the adherence of each thesis to the following criteria along with the indexing data:

1. MDGs
2. Faculty RPs
3. PHD RPs

This process was carried out and validated by two of the co-authors (Y.S.G, W.A.K) independently by screening all the titles, abstracts and summaries of the theses and also by continuous discussions throughout the study conduction. The whole process was finally revised and validated by the third author (M.I.E.).

### 2.5 Data Analysis

Data was entered and analyzed using the Statistical Package for Social Sciences Software (SPSS) version 16. Findings were presented as frequencies and percentages in relation to the different study variables as the type of thesis, time period and adherence to the national and international research priorities. The analysis plan targeted the assessment of differences between pre and post declaration of the international and national RP priorities. Suitable statistical tests were applied and results were considered significant at a P-value < 0.05.

### 2.6 Ethical Considerations

Requesting the hard and soft copies of the retrieved theses was done after obtaining all the administrative permissions from the Vice Dean Office for Postgraduate Affairs and the Chairman of the Post Graduate Library of the Faculty of Medicine, Cairo University.

## 3. Results

**Profile and trends of PH Research (1990-2014)**

The total number of theses produced by the PHD, Cairo University from 1990 till the end of 2014 was 245 theses distributed as 145 Master theses and 100 MD theses. The distribution of the theses according to the assigned three time periods of the study was as follows; 104 theses in 1990-2000, 88 theses in 2001-2010 and 53 theses in 2011-2014.

**Figure 1:**
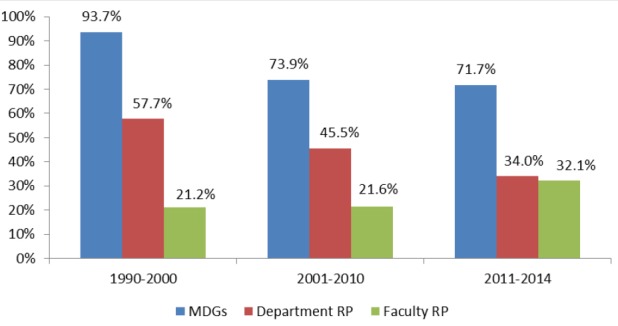
Profile of theses according to percent fulfillment of MDGs, Faculty and department research plans from 1990 to the end of 2014

The same trend was noticed in the first two periods where the highest commitment was to address the MDGs (93.7% in 1990-2000 and 73.9% in 2001-2010) followed by the department RP (57.7% in 1990-2000 and 45.5% in 2001-2010) and the least commitment was to address the Faculty RP (21.2 % in 1990-2000 and 21.6% in 2001-2010). This profile has changed in 2011-2014; despite the commitment to address the MDGs was still the highest (71.7%), there was nearly equal representation of thesis in relation to fulfillment of the PHD and Faculty RPs (34% and 32.1% respectively) (Figure 1).

No significant difference was detected between the size of research output in the form of Master and MD theses in addressing the MDGs, Faculty and department RPs in the three assigned study periods.

**Figure 2:**
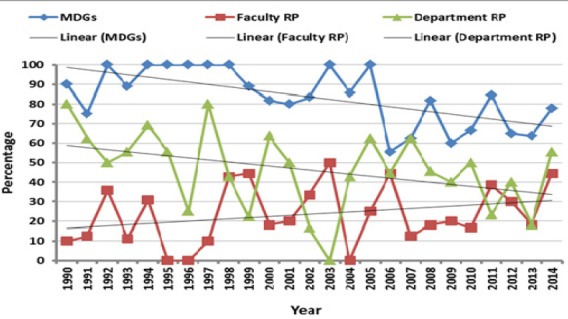
Trend lines for theses targeting MDGs, Faculty and department research plans in the time period 1990-2014 expressed as percentage of total theses for each year

The percent of theses fulfilling the MDGs reached the peak (100%) in years 1994-1998, 2003 and 2005. The percent of theses addressing the department RP was the highest (80%) in years 1990 and 1997, while the maximum compliance to the Faculty RP (50%) was in 2003. However, linear trend lines of the percent of theses fulfilling the MDGs and the department RP were declining and that of the Faculty was rising overtime (Figure 2).

The percent of theses fulfilling the MDGs reached the peak (100%) in years: 1992, 1994-1998, 2003 and 2005. The percent of theses addressing the department RP was the highest (80%) in years 1990 and 1997, while the maximum compliance to the Faculty RP (50%) was in 2003. However, linear trend lines of the percent of theses fulfilling the MDGs and the department RP were declining and that of the Faculty was rising overtime (Figure 2).

No significant difference was detected between the size of the research output in the form of Master and MD theses addressing the MDGs, Faculty and department RPs in the three assigned study periods.

Further analysis of compliance to the MDGs showed that the highest compliance was to address Goal 6 during the whole study period contributing to 34.6% of all the theses in 1990-2000, 46.6% in 2001-2010 and about half the theses (49.1%) in 2011-2014 (Table 1). However, most of the theses targeting Goal 6 were addressing diseases other than Human Immune Deficiency Virus (HIV)/AIDs, malaria and tuberculosis (T.B.). During the study period 1990-2014, HIV/AIDs, malaria and T.B research were represented only by 0.4%, 0% and 1.2% among thesis targeting Goal 6.

**Table 1:** Percent distribution of theses according to the MDGS and the time period

MDGs	1990-2000		2001-2010		2011-2014	
	No.	%	No.	%	No.	%
Goal 1: Eradicate extreme poverty and hunger	4	3.8	4	4.5		
Goal 2: Achieve universal primary education						
Goal 3: Promote gender equality and empower women	1	1				
Goal 4: Reduce child mortality rates	27	26	3	3.4	1	1.9
Goal 5: Improve maternal health	20	19.2	15	17	8	15.1
Goal 6:Combat HIV/AIDS, malaria, and other diseases	36	34.6	41	46.6	26	49.1
Goal 7: Ensure environmental sustainability	3	2.9				
Goal 8:Develop a global partnership for development	6	5.8	2	2.3	3	5.7
Fulfilling MDGs*	97	93.3	65	73.9	38	71.7
Not fulfilling MDGs*	7	6.7	23	26.1	15	28.3
**Total**	104	100	88	100	53	100

* P< 0.001.

Theses targeting Goal 4 contributed to nearly quarter the theses (26%) in 1990-2000 but this was followed by marked drop to 3.4% in 2001-2010 and 1.9% in 2011-2014. Meanwhile, the percent of theses addressing Goal 5 was more or less steady during the study period ranging from 15.1% in 2011-2014 to 19.2% of the total number of theses in 1990-2000. Table (1) also illustrates the absence of representation of Goals 2, 3 and 7 during the periods 2001-2010 and 2011-2014. Goal 1 was represented by 8 theses during the whole study period; representing 3.8% of the theses in 1990-2000 and 4.5% in 2001-2010. All the theses addressing Goal 1 in the current study were tackling the underweight among the under-five years of age children.

**Compliance and Gaps to National Health Priorities**

In the current study, national health priorities were represented by the Faculty and department RP priorities. The Faculty RP priorities enlisted the most prevalent and challenging communicable and non-communicable diseases in Egypt namely hepatitis, cancer, cardiovascular diseases, diabetes, obesity and parasitic infestations. No significant differences were found between PH theses addressing the Faculty RPs and those which were not before and after launching the Faculty RP in 2010 (P=0.266) (Table 2). In 1990-2000; theses addressing parasitic infections had the highest contribution among all PH theses (8.7%). However, in 2001-2010; there was a shift towards cancer and hepatitis research (6.8% each). In 2011-2014, cancer, obesity and diabetes research occupied the top three ranks among all PH theses by 11.3%, 7.5% % and 5.7%, respectively.

**Table 2:** Percent distribution of theses according to the Faculty research plan and the time period

Faculty RP	1990-2000		2001-2010		2011-2014	

	No.	%	No.	%	No.	%
**Hepatitis A,B,C**			6	6.8	1	1.9
**Cancer**	4	3.8	6	6.8	6	11.3
**Hypertension**					1	1.9
**Diabetes**	2	1.9	2	2.3	3	5.7
**Hypertension & diabetes**					1	1.9
**Obesity**	3	2.9	1	1.1	4	7.5
**Heart diseases**	4	3.8	2	2.3	1	1.9
**Parasitic diseases**	9	8.7	2	2.3		
Fulfilling Faculty RP*	22	21.2	19	21.6	17	32.1
Not fulfilling Faculty RP*	82	78.8	69	78.4	36	67.9
**Total**	104	100	88	100	53	100

* P=0.266.

Reproductive health research together with hepatitis, cancer and renal diseases were the main research priority areas launched by the PHD in 2010. Theses fulfilling the PHD RP represented 57.7% of all theses in the period from 1990 till 2000, 45.5% of theses in 2001-2010; meanwhile there was a drop after launching the PHD RP to reach 17% in 2011-2014. These differences were statistically significant at P=0.016 (Table 3). RH research had the highest contribution among all theses in addressing the PHD RP as follows: 53%, 29.5% and 17% in the periods 1990-2000, 2001-2010 and 2011-2014, respectively. This was followed by cancer research which represented 11.3% of all theses in 2011-2014. Research in renal diseases and hepatitis was the least represented after 2011 by 3.8% and 1.9%, respectively.

**Table 3:** Percent distribution of theses according to the Public Health department research plan and the time period

Department RP	1990-2000		2001-2010		2011-2014	
		
	No.	%	No.	%	No.	%
**Hepatitis**	0	0	6	6.8	1	1.9
**Cancer**	4	3.8	6	6.8	6	11.3
**Renal diseases**	1	1	2	2.3	2	3.8
**Reproductive Health**	55	52.9	26	29.5	9	17
Fulfilling Department RP*	60	57.7	40	45.5	18	34
Not fulfilling Department RP*	44	42.3	48	54.5	35	66
**Total**	104	100	88	100	53	100

* P=0.016.

## 4. Discussion

In the current study, compliance of academic researchers to the international and national research priorities was not satisfactory mainly due to the absence of proper monitoring and evaluation tools for assessing research output especially the theses which are considered as grey literature; being difficult to locate and acquire.

The MDGs are the most widely supported and comprehensive goals the world has ever established as they provided a definite framework for facing poverty, hunger, maternal and child health, communicable diseases, education, gender inequality and the global partnership for development (Lomazzi, Borisch, & Laaser, 2014). Proper monitoring and evaluation of MDGs achievements and failures is crucial for policy decisions, allocating funds and taking corrective interventions (Bourguignon et al., 2008).

As regards the research theses’ profile in the current study, addressing the MDGs received the highest priority throughout the whole assigned study periods followed by the PHD and Faculty RPs. Linear trend lines illustrated that the peak representation of theses targeting the MDGs was both before (1994-1998) and after (2003 and 2005) their launching. This finding could be a reflection that the majority of PH professionals in the Faculty of Medicine, Cairo University approved the MDGs and looked optimistically on their achievability despite the considerable challenges present in a developing country like Egypt. Similarly, in another study conducted by the World Federation of Public Health Associations (WFPHA), most of the PH professionals approved the MDGs despite the discrepancy of perceived importance of different goals (WFPHA, 2014).

In this study, the percent of PH research theses addressing the MDGs was significantly higher (P<0.001) in all the assigned study periods compared to those which were not, however, there was nearly a 20% decline after launching the MDGs in 2000 which could be attributed to a shift in community health needs at that period. Although all the MDGs are relevant to the health sector, three goals focus specifically on its performance namely reducing infant and child mortality (Goal 4), improving RH (Goal 5) and slowing the progress of HIV/AIDS, TB, malaria and other major diseases (Goal 6), especially in developing countries (Lomazzi et al., 2014; Travis et al., 2004). In accordance, compliance to MDGs 4, 5 and 6 was obvious in the current study as they were more represented than others before and after launching the MDGs in 2000 except Goal 4 which was less represented by PH theses after 2000. However, gaps were found in which there was no representation of Goals 2 (Achieving universal primary education), 3 (Promoting gender equality and empowering women) and 7 (Ensuring environmental sustainability) after launching the MDGs and also no representation of Goal 1 (Eradicating extreme poverty and hunger) in the period from 2011 till 2014. These findings could be justified by the nature of work of PH researchers as they are mainly involved in research related to MDGs 4, 5 and 6 in their day to day activities in the Faculty of Medicine, Cairo University. However, gaps in research addressing MDGs 1, 2, 3 and 7 could be a result of their perception that the underlying causes addressed in them are not under their direct influence and require extensive resources and inter-sectorial co-operation at the political and organizational level. Similarly, in a study conducted by Lomazzi et al. (2013) to explore the opinion of PH professionals worldwide and their experience regarding the implementation and achievement of MDGs, they stated that they are more involved in their daily work in research they are able to influence namely MDGs 4, 5 and 6.

In general, PH research in Egypt reflects the local health needs of the community represented by the official health indicators and reports produced regularly by governmental and non-governmental organizations e.g. Egyptian Demographic and Health Survey (EDHS), Central Agency for Public Mobilization and Statistics (CAPMAS) and Ministry of Health and Population (MOHP). Regarding MDGs 4 and 5 in the current study, they were more represented by PH theses before year 2000. This could be attributed to the country’s strong focus to reduce child and maternal mortality in that period (World Bank, 2014) which was reflected on the quantity of research conducted. In 1990, high under-five mortality rate reaching 104 per 1000 (United Nations Development Program [UNDP], 2010) has stimulated national programs to support child health as demonstrated by improved immunization coverage and access to safe water and control of acute respiratory infections and diarrheal diseases. The MOHP has adopted the Integrated Management of Childhood Illness (IMCI) strategy in 1997 as a comprehensive approach to improve the quality of primary health care services offered to children, and it has been associated with doubling the reduction rate of under-five mortality in districts where it has been implemented (MOHP, 2014). In the present study, the marked reduction observed in representation of Goal 4 by PH theses after 2000 could be related to improvement in child mortality rates where it declined to 54 per 1000 in year 2000 according to the EDHS and continued declining to reach 28.3 in 2008, which is considered the MDG target for 2015 (UNDP, 2010).

As regards Goal 5 which is reducing by three quarters the maternal mortality ratio (MMR) and achieving universal access to RH, trends of MMR in Egypt have shown significant improvement from 174 per 100,000 live births in 1992 to reach 84 in 2000 (Gipson, Campbell, Issa, Matta, & Mansour, 2005) and about 55 in 2008, which is the MDG target for 2015 (World Bank, 2014). These findings represented about 50% reduction during 1992-2000 and about 70% reduction in the later period to 2008 (UNDP, 2010) which was associated with high rates of family planning use, improved antenatal care coverage and skilled birth attendance (El-Saharty, Richardson, & Chase, 2005). Although there has been marked improvement on the national level regarding MMR, this was not accompanied by a parallel reduction in PH theses addressing maternal and RH in the current study. This could be justified by the importance of these as priority research areas in the PHD research plan and the persistence of challenges facing maternal health e.g. management of maternal hemorrhage. Despite that institutional deliveries in Egypt have increased by more than 80% between 1992 and 2000; blood shortage in 2000 was still among the most frequent avoidable health facility factors for reducing maternal mortality (Gay et al., 2004).

In the current study, the highest compliance was for MDG 6 with more representation after 2000, but with addressing diseases other than HIV/AIDS, malaria and TB. Egypt has showed marked progress towards eradication of Malaria and TB; two of the major health challenges for the new millennium. The incidence of malaria has declined from four cases to almost Zero per 1000 between 1990 and 2000 (Ministry of Economic development, 2008). As regards TB, Egypt has achieved the global targets in case detection and treatment success under the Directly Observed Treatment Short Course (DOTS) strategy (Alwani, Al-Aarag, Omar, & Hibah, 2015) where the case detection rate of positive cases in 2008 was 78% (global target was 70%) and treatment success rate was 88% (global target was 85%) (UNDP, 2010). Hence, there was a reflection on the quantity of PH research targeting these diseases as follows: no theses targeting malaria at all and only one thesis targeting TB in the period from 2001-2010.

In Egypt, there are no precise estimates to detect HIV prevalence and thus it is very difficult to assess the HIV epidemic status in the country (UNDP, 2010) and to achieve universal access to treatment for all those who need it. Until the end of 2009, there were 3919 HIV infected cases detected, however, higher estimates were provided by the UNAIDS/WHO reaching 10,200 till the year 2008 (MOHP, 2009). The under estimation in the national statistics is among the challenges facing national efforts in Egypt to combat HIV together with the wide range of HIV transmission routes and the severe stigmatization towards people living with HIV (National AIDS Program, 2009). Despite the need for more PH research to resolve the challenges facing HIV interventions in Egypt, difficulties exist due to the conservative nature of the disease and this was reflected by the gap in addressing HIV research in the current study where only one PH thesis after 2010 targeted it.

One of the Faculty plan objectives during the period from 2010-2014 was that 80% of community-based research should be directed towards major and challenging diseases namely hepatitis, cancer, hypertension, diabetes, obesity, heart diseases and parasitic infestations [14]. However, in the current study, only about 32% of PH theses after 2010 were directed towards these areas with slight increase (nearly 10%) compared to those conducted before 2010. This could be attributed to lack of co-ordination and communication between policy-makers in the Faculty and researchers in different departments including PHD.

Before 2000, theses targeting parasitic infestations had the highest representation (8.7%); however, they declined to 2.3% in the period from 2001 till 2010. This could be attributed to the high prevalence of Schistosomiasis in Egypt in 1993 reaching 14.8% for intestinal Schistosomiasis which dropped to 0.4% in 2009 (Barakat, 2013). In the period from 2001-2010, there was a shift in research theses’ trends towards cancer and hepatitis being among the major health threats and leading causes of death in Egypt. Rates of HCV infection in Egypt are higher than neighboring countries, as well as other countries in the World, where the EDHS conducted in 2008 estimated HCV prevalence among the 15-59 years age to be 14.7% (El-Zanaty & Way, 2008). Moreover, unsafe practices and stigma towards the at-risk groups are among the challenges facing Egypt to combat this disease (Guerra, Garenne, Mohamed, & Fontanet, 2012).

From 2011 till 2014, there was a remarkable shift in the theses addressing the Faculty RPs towards non-communicable diseases namely cancer (11.3%), obesity (7.5%) and diabetes (5.7%). This shift could be related to the growing burden of non-communicable diseases especially in developing countries like Egypt which is still struggling to meet the challenges of existing problems of infectious diseases (Marshall, 2004). According to the WHO, non-communicable diseases including cardiovascular diseases, diabetes, cancer and obesity now account for 59% of the 56.5 million deaths that occur globally every year and almost half of the global burden of disease (Marshall, 2004).

Unexpectedly, PH research theses not fulfilling the department RPS were significantly higher (P=0.016) than those fulfilling them before (54.5% vs. 45.5%) and after (66% vs. 34%) their launching in 2010. This could be due to lack of communication channels between decision makers in the PHD and researchers and also absence of a proper research system in the PHD to set regulations in conducting research; to provide funds; and finally to set priorities for health research. Despite the considerable progress that Egypt has made concerning universal access to RH; challenges still exit to keep the gains; among these is the absence of a comprehensive package for RH and the regional disparities in delivery and utilization of maternal health care, particularly in Upper Egypt (UNDP, 2010). As a reflection of the previously mentioned facts and being one of most important PH domains, RH research theses had the highest rank in targeting the PHD RPs throughout the study periods.

### 4.1 Conclusion and Recommendation

After launching the MDGs in 2000 and the Faculty and department health RPs in 2010, the compliance of the academic researchers to respond to these international and national priorities was not satisfactory. There was a definite decline in research output tackling the MDGS and PHD research priorities, with a non-significant increase in the production of theses addressing the Faculty RPs. This may be attributed to many reasons concerning the local health needs and the research process itself. However, one of the most important factors is the absence of monitoring and evaluation tools for assessment of the academic research output especially the postgraduate theses. This study is a practical model for policy makers within the universities to develop and implement a reliable monitoring and evaluation system for assessing research output in relation to national and international research priorities. In addition, more studies should be initiated to assess the qualitative aspects of the academic research output.

**Abbreviations**

MDGs: Millennium Development Goals; PH: Public Health; PHD: Public Health Department; RPs: research plans; UN: United Nations; RH: reproductive health; MD: Medical Doctoral; HIV: Human Immune Deficiency Virus; TB: Tuberculosis; EDHS: Egyptian Demographic and Health Survey; CAPMAS: Central Agency for Public Mobilization and Statistics; MOHP: Ministry of Health and Population; IMCI: Integrated management of Childhood Illness; MMR: maternal mortality ratio; DOTS: Directly Observed Treatment Short Course.

## References

[ref1] Alwani A, Al-Aarag A, Omar M, Hibah N (2015). Incidence of tuberculosis before and after DOTS (direct observed therapy short course strategy) implementation in El-Behira Governorate, Egypt. Egypt J Bronchol.

[ref2] Barakat R (2013). Epidemiology of Schistosomiasis in Egypt: Travel through Time: Review. J Advanced Research.

[ref3] Bourguignon F, Benassy-Quere A, Dercon S, Estache A, Gunning J. W, Ravikanbur R (2008). Millennium Development Goals at midpoint: where do we stand and where do we need to go?.

[ref4] Devex and United Nations Foundation (2015). Making the Millennium Development Goals happen. Perspectives from Global Aid Workers & Development Professionals on achieving the MDGs.

[ref5] El-Saharty S, Richardson G, Chase S (2005). Egypt and the Millennium Development Goals: Challenges and Opportunities. Discussion Paper by the Health, Nutrition, and Population Family (HNP) of the World Bank’s Human Development Network, 2005.

[ref6] El-Zanaty F, Way A (2008). Egypt Demographic and Health Survey 2008.

[ref7] Faculty of Medicine Cairo University (2011). Kasr Al-Ainy New Vision for a New Era, Faculty of Medicine, Cairo University.

[ref8] Fatokun J, Amusa O (2014). Acquisition and management of grey literature: A case study of the Federal University of Agriculture, Abeokuta, Nigeria. Chinese Librarianship: An International Electronic Journal.

[ref9] Gay J, Hardee K, Judice N, Agarwal K, Fleming K, Hairston A, Wood M (2003). What works: A policy and program guide to the evidence on family planning, safe motherhood, and STI/HIV/AIDS interventions. Policy Project: 2003.

[ref10] Gipson R, Campbell O, Issa A. H, Matta N, Mansour E (2005). The trend of maternal mortality in Egypt from 1992-2000: an emphasis on regional differences. Maternal and Child Health Journal.

[ref11] Guerra J, Garenne M, Mohamed M. K, Fontanet A (2012). HCV burden of infection in Egypt: Results from a nationwide survey. J Viral Hepatitis.

[ref12] LaPelle N, Luckmann R, Simpson E, Martin E (2006). Identifying strategies to improve access to credible and relevant information for PH professionals: A qualitative study. BMC PH.

[ref13] Lomazzi M, Borisch B, Laaser U (2014). The Millennium Development Goals: experiences, achievements and what’s next. Glob Health Action.

[ref14] Lomazzi M, Laaser U, Theisling M, Tapia L, Borisch B (2014). Millennium Development Goals: How public health professionals perceive the achievement of MDGs. Glob Health Action.

[ref15] Lomazzi M, Theisling M, Tapia L, Borisch B, Laaser U (2013). MDGs_a public health professional’s perspective from 71 countries. J Public Health Policy.

[ref16] Marshall S (2004). Developing countries face double burden of disease. B World Health Organ.

[ref17] McCarthy M, Harvey G, Conceicao C, la Torre G, Gulis G (2009). Comparing public-health research priorities in Europe. Health Research Policy and Systems.

[ref18] Millennium Development Goals (2015). In Wikipedia, the free encyclopedia.

[ref19] Ministry of Economic Development (2008). Egypt achieving the Millennium Development goals: A mid-point assessment.

[ref20] Ministry of Health and Population (2009). HIV/AIDS Surveillance Report.

[ref21] MOHP (2014). Ministry of Health and Population Egypt, Partnership for Maternal, Newborn & Child Health, WHO, World Bank and Alliance for Health Policy and Systems Research. Success factors for women’s and children’s health: Egypt.

[ref22] National AIDS Program (2009). Ungass Country Progress Report, Arab Republic of Egypt, January 2008-December 2009.

[ref23] Osayande O, Ukpebor C, Grey literature acquisition and management: Challenges in academic libraries in Africa (2012). Library Philosophy and Practice, paper 700.

[ref24] PROCOSI (2013). Civil society consultation on health in the post-2015 development agenda. Procosi.

[ref25] The World We Want (2013). World We Want.

[ref26] Travis P, Bennett S, Haines A, Pang T, Bhutta Z, Hyder A, Pielemeier N (2004). Overcoming health-systems constraints to achieve the Millennium Development Goals. Lancet.

[ref27] UNDP (2010). Egypt’s progress towards achieving the Millennium Development Goals.

[ref28] United Nations (2000a). Official list of MDG indicators.

[ref29] United Nations (2000b). United Nations millennium declaration.

[ref30] United Nations (2014). The Millennium Development Goals report 2014.

[ref31] WFPHA (2014). World Federation of Public Health Associations.

[ref32] WHO (2012). Informal member state consultation on health in the Post 2015 development agenda.

[ref33] WHO (2013). Health in the post-2015 UN development agenda.

[ref34] World Bank (2014). Achieving MDGs 4 & 5: Egypt’s progress on maternal and child health. Health, nutrition and population global practice knowledge brief, 1.

